# Comprehensive Comparison of Two Color Varieties of Perillae Folium by GC-MS-Based Metabolomic Approach

**DOI:** 10.3390/molecules27206792

**Published:** 2022-10-11

**Authors:** Jiabao Chen, Dan Zhang, Qian Wang, Aitong Yang, Yuguang Zheng, Lei Wang

**Affiliations:** 1 Traditional Chinese Medicine Processing Technology Innovation Center of Hebei Province, College of Pharmacy, Hebei University of Chinese Medicine, Shijiazhuang 050200, China; 2 International Joint Research Center on Resource Utilization and Quality Evaluation of Traditional Chinese Medicine of Hebei Province, Hebei University of Chinese Medicine, Shijiazhuang 050200, China; 3 Department of Pharmaceutical Engineering, Hebei Chemical and Pharmaceutical College, Shijiazhuang 050026, China

**Keywords:** perilla leaf, chemical composition, GC-MS, multivariate statistical analysis, biosynthetic pathway

## Abstract

Perillae Folium (PF), the leaf of *Perilla frutescens* (L.) Britt, is extensively used as culinary vegetable in many countries. It can be divided into two major varietal forms based on leaf color variation, including purple PF (*Perilla frutescens* var. *arguta*) and green PF (*P. frutescens* var. *frutescens*). The aroma of purple and green PF is discrepant. To figure out the divergence of chemical composition in purple and green PF, gas chromatography–tandem mass spectrometry (GC-MS) was applied to analyze compounds in purple and green PF. A total of 54 compounds were identified and relatively quantified. Multivariate statistical methods, including principal component analysis (PCA), orthogonal partial least-squares discrimination analysis (OPLS-DA) and clustering analysis (CA), were used to screen the chemical markers for discrimination of purple and green PF. Seven compounds that accumulated discrepantly in green and purple PF were characterized as chemical markers for the discrimination of the purple and green PF. Among these 7 marker compounds, limonene, shisool and perillaldehyde that from the same branch of the terpenoid biosynthetic pathway were with relatively higher contents in purple PF, while perilla ketone, isoegomaketone, tocopheryl and squalene on other branch pathways were higher in green PF. The results of the present study are expected to provide theoretical support for the development and utilization of PF resources.

## 1. Introduction

*Perilla frutescens* (L.) Britt. is an annual herbal plant that belongs to the family of Lamiaceae [1,2]. The leaf of *P. frutescens* (L.) Britt, also called Perillae Folium (PF), has been extensively used in many countries as a culinary vegetable. Based on plant leaf color variation, PF can be divided into two major varietal forms that are circulated in China, including purple PF *(P. frutescens* var. *arguta*) and green PF (*P. frutescens* var. *frutescens*) [3]. *P. frutescens* var. *arguta* and *P. frutescens* var. *frutescens* are considered the same species in plant taxonomy, but there are large differences in practical application. Purple PF is widely used as a natural food pigment and a genuine medicinal plant for the treatment of food poisoning, coughs and gastritis [4,5,6]. Purple PF is believed to have efficacy in exterior relief, dispersing cold, easing stomach pain, reducing phlegm and relieving coughs and asthma [7]. Traditionally, it has been used to alleviate a variety of symptoms, including coughs, colds, fever, allergies and some intestinal diseases [8,9]. Unlike purple PF, green PF is consumed only as a vegetable or industrial preservative and is not used as a traditional Chinese medicine in China [3].

Phytochemical studies indicate that PF is rich in volatile compounds [10,11,12], flavonoids [13,14], anthocyanins [15], fatty acids [16,17] and phenolic compounds [18,19]. Compounds and extractions of PF showed various biological activities, such as antioxidant, antimicrobial, antiallergic, antidepressant, anti-inflammatory and anticancer effects [20,21,22,23,24]. Metabolites in foods or natural herbs differ by varietal forms, which may produce effects on their quality and effectiveness. Therefore, it is necessary to clarify the chemical differences of different PF. Huang et al. [25] compared the content and composition of the volatiles of purple and green PF, obtained by SFE, HS-SPME and hydrodistillation. A total of 64 volatile compounds were identified in purple and green PF by GC-MS, with 29 components simultaneously found in both of them. Tabanca et al. [26] identified 27 volatile compounds in purple and green PF by GC-MS, with only 8 compounds present simultaneously in both of them. Fan et al. [27] reported that a total of 57 nonvolatile chemical components and 105 volatile chemical components were characterized in leaves, stems and seeds of different varieties of perilla by ultrahigh-performance liquid chromatography coupled with quadrupole time-of-flight mass spectrometry (UPLC-Q-TOF-MS/MS) and GC-MS. Furthermore, 27 nonvolatile constituents and 16 volatile constituents were identified as potential markers for discriminating perilla between different varieties. Deguchi et al. [28], using high-performance liquid chromatography (HPLC), reported that the main phenolic compound rosmarinic acid content was higher in green PF compared with purple PF. Zheng et al. [29] investigated the difference in the chemical compositions between green PF and purple PF by rapid resolution liquid chromatography coupled with quadruple time-of-flight mass spectrometry (RRLC-Q/TOF-MS), and revealed that flavonoids and anthocyanins in particular had higher contents in purple PF. Additionally, their results showed that the purple PF had more pronounced antioxidative activities than the green PF.

In the present study, purple PF and green PF were compared and distinguished from the aspect of chemical composition by the GC-MS-based metabolomic approach. In addition, multivariate statistical methods, including principal component analysis (PCA), orthogonal partial least-squares discrimination analysis (OPLS-DA) and clustering analysis (CA) were used to screen the chemical markers between purple and green PF.

## 2. Results and Discussion

### 2.1. Compounds Identification

**Figure 1:**
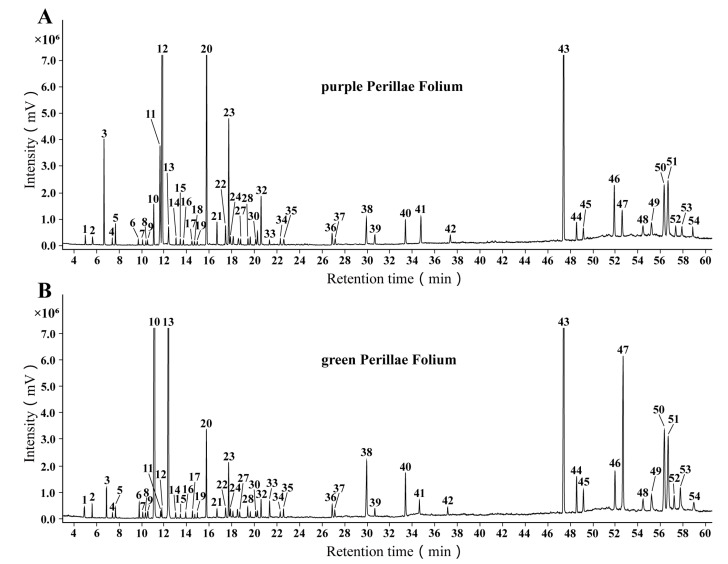
The typical total ion chromatograms of n-hexane extracts of (**A**) purple Perillae Folium and (**B**) green Perillae Folium by GC-MS. The number of peaks was consistent with those of compounds in Table 2.

In this study, the chemical profiling of n-hexane extract in 12 batches of purple PF and 10 batches of green PF (sample information see in Table 1) was achieved by GC-MS. The representative total ion chromatogram (TIC) of the two varietal forms of PF is shown in Figure 1. With reference to the NIST17 database, 54 compounds were identified by comparing their mass spectra. Most of the identified compounds belong to monoterpenes and sesquiterpenes. The retention time, retention index, molecular weight and molecular formula of the identified compounds are summarized in Table 2.

### 2.2. Chemical Comparison of Purple and Green PF

In this work, all 54 detected compounds were found in both purple and green PF, with their contents varying. To further specify the difference of the n-hexane extract profiles of purple and green PF, multivariate statistical methods, including PCA, OPLS-DA and CA, were used to analyze the data.

**Figure 2:**
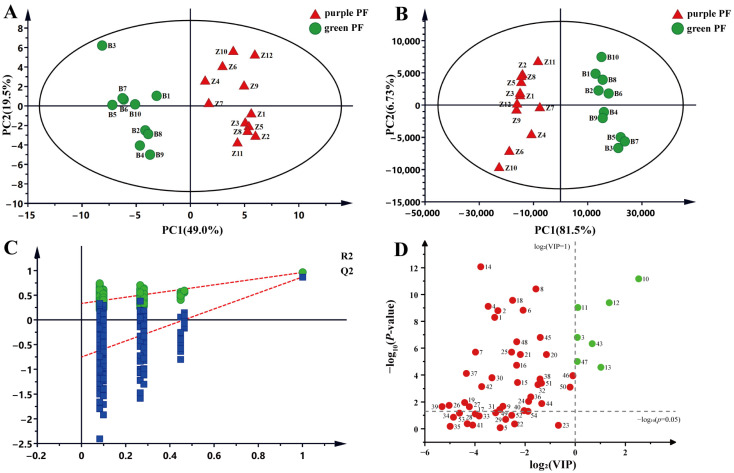
Determination of differential compounds from two PF varieties. (**A**) Unsupervised PCA score plot of purple and green PF samples. PC1 occupies 49.0% and PC2 19.5% of total variance. (**B**) Supervised OPLS-DA score plot of purple and green PF samples. PC1 occupies 81.5% and PC2 6.73% of total variance. (**C**) Permutation test at 200 times used for the discrimination between the two PF varieties. (**D**) Scatter plot of *p*-value and VIP value. The green points show differential compounds with VIP > 1, *p* < 0.05.

PCA is an unsupervised pattern recognition method to visualize grouping trends and outliers. PCA was performed with 54 compounds used as independent variables. As shown in the PCA scores plot (Figure 2A), all samples were clearly separated into two groups corresponding to purple PF and green PF. The first two components explained 68.5% of the total variance. PCA results indicated that the purple PF and green PF samples were indeed different in terms of the content of identified compounds.

OPLS-DA is a supervised pattern recognition method that can be used to analyze, classify and reduce the dimensionality of complex datasets. To filter out the differential components of the two varietal forms of PF, the GC-MS data were analyzed by OPLS-DA. The OPLS-DA scores plot (Figure 2B) shows that purple PF and green PF can also be clearly classified into two groups. Further to validate the model of OPLS-DA, a permutation test (*n* = 200) was conducted. The results of R2Y (cum) = 0.962 and Q2 (cum) = 0.870 (Figure 2C), indicated good classification and predictability of the OPLS-DA model. By using the metabolite features with VIP > 1 and *p* < 0.05, 7 compounds, including D-limonene (3), perilla ketone (10), shisool (11), perillaldehyde (12), isoegomaketone (13), squalene (43) and tocopheryl (47), were screened out as potential chemical markers for distinguishing purple PF and green PF (Figure 2D). The relative peak areas (%) of potential chemical markers in purple and green PF were calculated (Table 3). The results indicated that perilla ketone (10) was the most abundant compound in green PF, with relative peak areas of 27.50 ± 3.01%, while perillaldehyde (12) was the most abundant compound in purple PF, with relative peak areas of 31.72 ± 3.12%.

**Figure 3:**
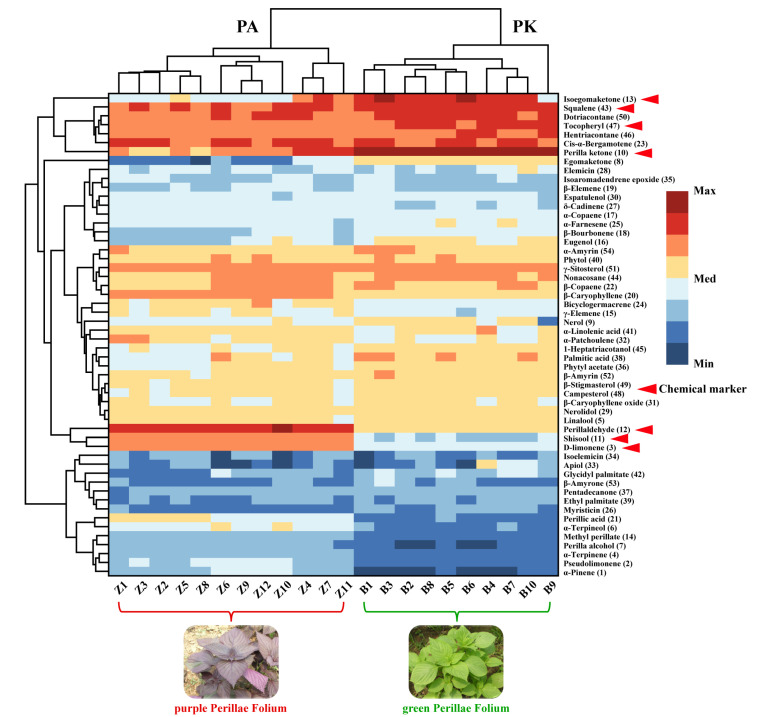
The relative concentration trends of identified compounds in purple PF and green PF.

CA is a multivariate statistical method to classify samples or indicators, and a heatmap was used to show the relative concentration trends of compounds across all samples. In order to visualize the differences in metabolic profiles between the two varieties of PF, the peak areas of 54 compounds were used to construct a heatmap. The heatmap (Figure 3) showed that the two PF varieties could be clearly distinguished on the basis of the clustering relationships of the identified compounds, consistent with the results of PCA and OPLS-DA. Among the 54 compounds, the content of perillaldehyde (12), shisool (11), D-limonene (3), perillic acid (21), α-terpineol (6), perilla alcohol (7), terpinene (4) and α-pinene (1) in purple PF was significantly higher than that of green PF, while isoegomaketone (13), squalene (43), dotriacontane (50), tocopheryl (47), hentriacontane (46), perilla ketone (10) and perilla ketone (8) had higher content in green PF. Specifically, the main identified components in purple and green PF were perillaldehyde (12) and perilla ketone (10), respectively. According to the classification principles of volatile oil chemotypes of PF in previous studies [30,31], all purple PF samples of volatile oil chemotypes were PA (perillaldehyde), and all green PF samples were PK (perilla ketone).

**Figure 4:**
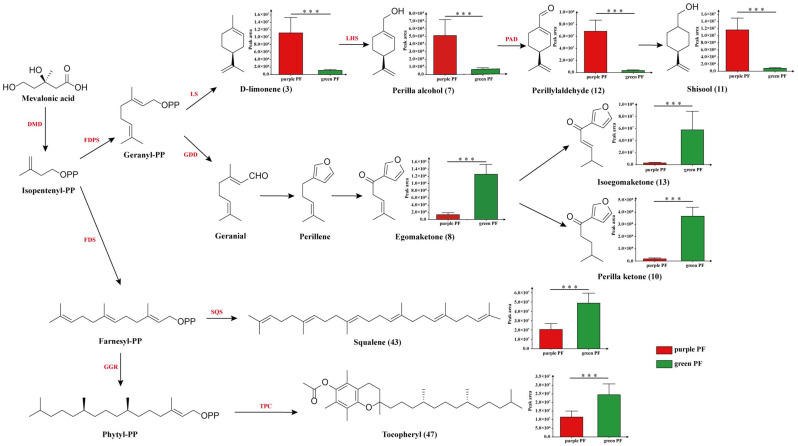
Putative biosynthetic pathways of the main terpenoids in perilla. Metabolites are written in black letters, whereas enzymes are written in red letters. DMD, diphosphomevalonate decarboxylase; FDPS, farnesyl diphosphate synthase; LS, limonene synthase; LHS, limonene hydroxylase; PAD, perillylalcohol dehydrogenase; GDD, geranyl diphosphate diphosphohydrolase; FDS, farnesyl diphosphate synthase; SQS, squalene synthase; GGR, geranylgeranyl reductase; TPC, tocopherol C-methyltransferase. *** *p* < 0.001.

Considering the biosynthetic information of potential chemical markers, perilla alcohol (7), shisool (11) and perillaldehyde (12) that metabolized from limonene (3) all had higher contents in purple PF samples, whereas egomaketone (8), perilla ketone (10) and isoegomaketone (13) that derived from geranial together with squalene (43) and tocopheryl (16) had higher contents in green PF (Figure 4).

Generally, the potential mechanisms of differences in chemical composition are related with genes encoding biosynthetic enzymes and regulatory proteins [32]. Zheng et al. [29] reported that the conserved gene sequences of ITS2 (internal transcribed spacer 2) are consistent in green and purple PF, which suggests that it is reasonable to classify them as the same species of *P. frutescens* (L.) Britt from the perspective of plant taxonomy. Therefore, the obvious differences in the chemical composition between the two varieties of PF may relate with nonconserved gene regions and downstream regulatory proteins. Previous research had found quite different levels of the PFLC1 gene encoding limonene synthase in different perilla chemotypes [33]. The content difference of identified terpenoids in purple and green PF might be related with the expression of key genes encoding limonene synthase (LS), geranyl diphosphate diphosphohydrolase (GDD) and farnesyl diphosphate synthase (FDS).

## 3. Materials and Methods

### 3.1. Plant Material

A total of 12 batches of purple PF (*P. frutescens* var. *arguta*) and 10 batches of green PF (*P. frutescens* var. *frutescens*), were collected from Hebei Academy of Agriculture and Forestry Sciences in Shijiazhuang (China 38°06′41.7″ N, 114°45′35.8″ E) on 30 August 2019 and identified by Yuguang Zheng, professor in the field of identification of Chinese Medicine. The origins of the 22 samples are listed in Table 1. The harvested leaves were air-dried in the dark at room temperature for 2 weeks to acquire consistently low water content. All voucher specimens were deposited in dry, dark room of Traditional Chinese Medicine Processing Technology Innovation Center of Hebei Province, Hebei University of Chinese Medicine with their specimen number (see Table 1).

### 3.2. Metabolite Extraction

Plant materials of each batch were pulverized and screened through 60-mesh sieves. The powdered sample was extracted according to an ultrasonic extraction protocol [34] with some modification. A total of 0.1 g of the powdered sample was extracted with 1 mL of n-hexane by means of sonication (power, 300 W; frequency, 40 kHz) for 15 min at room temperature. The extract was then centrifuged at 13,000 rpm for 10 min at room temperature. A total of 1μL of supernatant was injected into the GC-MS for analysis.

### 3.3. GC-MS Analysis

The GC-MS analysis was performed with an Agilent 7890B GC coupled with 5977B MSD mass detector (Agilent Technologies, Santa Clara, CA, USA). The GC-MS instrument coupled with an Agilent HP-5MS 5% phenyl methyl siloxane capillary column (30 m × 0.25 mm, 0.25 μm film thickness, Agilent, Santa Clara, CA, USA). Helium (≥99.999%) was used as carrier gas at a constant flow rate of 1.0 mL·min^−1^. A total of 1 μL of the prepared supernatant solution was injected in split mode with the split ratio set to 2:1 at a temperature of 250 °C. The oven temperature program was initially set at 45 °C, then raised to 100 °C at a rate of 10 °C·min^−1^ and subsequently raised to 280 °C at a rate of 4 °C·min^−^^1^, then finally held for 10 min. The quadrupole mass detector was operated in electron impact (EI) mode at 70 eVwith a mass range of 50–500 *m*/*z*. A total of 22 batches of samples were randomly analyzed with three replicates to ensure system stability throughout the analysis. n-Alkane standard solution (C_8_–C_20_, 40 mg·L^−1^, Sigma-Aldrich, Buchs, Switzerland) was analyzed under the same condition for retention index (RI) calculation.

### 3.4. Data Processing and Statistical Analysis

The identification of metabolites in purple and green PF were achieved by comparing the obtained mass spectra with reference mass spectra from the National Institute of Standards and Technology 17 (NIST17) library. The peaks in all the samples were aligned and matched by using Agilent MassHunter analysis program (Agilent, Santa Clara, CA, USA). The RI of all the identified compounds was calculated by comparing their corresponding peak retention time to that of n-alkanes (C8–C20) [35,36]. Finally, the resulting data matrix consisting of sample codes, variables and peak areas was extracted and used for statistical analysis.

The obtained data matrix was imported into SIMCA P13 software (Umetrics, Umea, Sweden) for principal component analysis (PCA) and orthogonal partial least-squares discrimination analysis (OPLS-DA). Cluster analysis (CA) was performed with Origin Pro 2020 (OriginLab Corporation, Northampton, MA, USA) software. *p*-value was calculated by independent-samples *t*-test with IBM SPSS Statistics 23.0 (IBM, Armonk, NY, USA) software.

## 4. Conclusions

In this study, a GC-MS-based metabolomics method for rapid discrimination of differential metabolites between purple and green PF was established. The chemical compositions of n-hexane extracts of purple and green PF were investigated and a total of 54 compounds were identified by comparison of their mass spectra with NIST17 library. Among them, 7 differential compounds between the two varieties of PF were screened and characterized using multivariate statistical methods and heatmap visualization analysis. The results indicated that purple PF and green PF samples could be distinguished from each other according to the relative content of these marker compounds. This study may offer data support for research and exploitation of purple and green PF, and provide a feasible method for the authentication of purple and green PF.

## Figures and Tables

**Table 1:** The information of collected purple Perillae Folium (Z1–Z12) and green Perillae Folium (B1–B10).

No.	Source	Specimen No.	No.	Source	Specimen No.
Z1	Hebei Province	PF201908Z01	Z12	Imported from Japan	PF201908Z12
Z2	Hebei Province	PF201908Z02	B1	Gansu Province	PF201908B01
Z3	Hebei Province	PF201908Z03	B2	Gansu Province	PF201908B02
Z4	Guizhou Province	PF201908Z04	B3	Gansu Province	PF201908B03
Z5	Hebei Province	PF201908Z05	B4	Hebei Province	PF201908B04
Z6	Hebei Province	PF201908Z06	B5	Gansu Province	PF201908B05
Z7	Hebei Province	PF201908Z07	B6	Hebei Province	PF201908B06
Z8	Hebei Province	PF201908Z08	B7	Gansu Province	PF201908B07
Z9	Sichuan Province	PF201908Z09	B8	Gansu Province	PF201908B08
Z10	Shanxi Province	PF201908Z10	B9	Gansu Province	PF201908B09
Z11	Gansu Province	PF201908Z11	B10	Liaoning Province	PF201908B10

**Table 2:** The information of the compounds in purple PF and green PF by GC-MS.

Peak No.	Retention Time (min)	Compounds	Molecular Weight	Molecular Formula	Retention Index	VIP	*p*-Value
1	5.01	α-Pinene	136	C_10_H_16_	918	0.11	***
2	5.66	Pseudolimonene	136	C_10_H_16_	964	0.12	***
3	6.45	D-limonene	136	C_10_H_16_	1018	1.02	***
4	7.52	α-Terpinene	136	C_10_H_16_	1083	0.09	***
5	7.69	Linalool	154	C_10_H_18_O	1093	0.13	-
6	9.71	α-Terpineol	154	C_10_H_18_O	1193	0.24	***
7	10.01	Perilla alcohol	152	C_10_H_16_O	1207	0.06	***
8	10.1	Egomaketone	166	C_10_H_14_O_2_	1210	0.34	***
9	10.54	Nerol	154	C_10_H_18_O	1229	0.14	*
10	11.21	Perilla ketone	166	C_10_H_14_O_2_	1257	5.78	***
11	11.71	Shisool	154	C_10_H_18_O	1277	1.06	***
12	11.87	Perillaldehyde	150	C_10_H_14_O	1284	2.56	***
13	12.45	Isoegomaketone	164	C_10_H_12_O_2_	1307	2.03	***
14	13.28	Methyl perillate	180	C_11_H_16_O_2_	1339	0.07	***
15	13.43	γ-Elemene	204	C_15_H_24_	1344	0.20	***
16	13.94	Eugenol	164	C_10_H_12_O_2_	1363	0.20	***
17	14.51	α-Copaene	204	C_15_H_24_	1385	0.06	-
18	14.77	β-Bourbonene	204	C_15_H_24_	1395	0.18	***
19	14.94	β-Elemene	204	C_15_H_24_	1401	0.05	*
20	15.77	β-Caryophyllene	204	C_15_H_24_	1431	0.45	***
21	16.66	Perillic acid	166	C_10_H_14_O_2_	1464	0.22	***
22	17.44	β-Copaene	204	C_15_H_24_	1492	0.19	-
23	17.74	Cis-α-Bergamotene	204	C_15_H_24_	1503	0.63	-
24	17.88	Bicyclogermacrene	204	C_15_H_24_	1508	0.28	**
25	18.09	α-Farnesene	204	C_15_H_24_	1516	0.17	***
26	18.51	Myristicin	192	C_11_H_12_O_3_	1531	0.03	*
27	18.59	δ-Cadinene	204	C_15_H_24_	1532	0.05	*
28	19.43	Elemicin	208	C_12_H_16_O_3_	1565	0.05	-
29	19.64	Nerolidol	222	C_15_H_26_O	1572	0.15	-
30	20.12	Espatulenol	220	C_15_H_24_O	1590	0.10	***
31	20.27	β-Caryophyllene oxide	220	C_15_H_24_O	1595	0.11	-
32	20.59	α-Patchoulene	204	C_15_H_24_	1607	0.36	***
33	21.35	Apiol	222	C_12_H_14_O_4_	1636	0.07	-
34	22.16	Isoelemicin	208	C_12_H_16_O_3_	1666	0.03	-
35	22.62	Isoaromadendrene epoxide	220	C_15_H_24_O	1683	0.03	**
36	26.89	Phytyl acetate	338	C_22_H_42_O_2_	1849	0.29	**
37	27.04	Pentadecanone	268	C_18_H_36_O	1855	0.05	***
38	29.91	Palmitic acid	256	C_16_H_32_O_2_	1973	0.38	***
39	30.67	Ethyl palmitate	284	C_18_H_36_O_2_	2005	0.03	*
40	33.39	Phytol	296	C_20_H_40_O	2119	0.24	*
41	34.89	α-Linolenic acid	278	C_18_H_30_O_2_	2181	0.06	-
42	37.32	Glycidyl palmitate	312	C_19_H_36_O_3_	2283	0.08	***
43	47.41	Squalene	410	C_30_H_50_	2705	1.60	***
44	48.56	Nonacosane	408	C_29_H_60_	2754	0.40	*
45	49.16	1-Heptatriacotanol	537	C_37_H_76_O	2779	0.38	***
46	51.91	Hentriacontane	436	C31H64	2894	0.98	***
47	52.61	Tocopheryl	430	C_29_H_50_O_2_	2923	1.01	***
48	54.44	Campesterol	400	C_28_H_48_O	3000	0.20	***
49	55.2	β-Stigmasterol	412	C_29_H_48_O	3031	0.12	*
50	56.33	Dotriacontane	450	C_32_H_66_	3079	0.87	***
51	56.68	γ-Sitosterol	414	C_29_H_50_O	3093	0.39	***
52	57.42	β-Amyrin	426	C_30_H_50_O	3124	0.17	-
53	57.98	β-Amyrone	424	C_30_H_48_O	3148	0.04	-
54	58.7	α-Amyrin	426	C_30_H_50_O	3178	0.27	-

“-” represent no significant difference. VIP, variable importance in projection. * *p* < 0.05; ** *p* < 0.01; *** *p* < 0.001.

**Table 3:** The relative peak areas (%) of the potential chemical markers in purple PF and green PF.

No.	Retention Time (min)	Retention Index	Compounds	Purple PF ( $\bar{X}$ ± SD, *n* = 12, %)	Green PF ( $\bar{X}$ ± SD, *n* = 10, %)
3	6.45	1018	D-limonene	5.12 ± 1.23	0.20 ± 0.04
10	11.21	1257	Perilla ketone	2.15 ± 0.97	27.50 ± 3.01
11	11.71	1277	Shisool	5.41 ± 0.86	0.05 ± 0.02
12	11.87	1284	Perillaldehyde	31.72 ± 3.12	0.60 ± 0.21
13	12.45	1307	Isoegomaketone	0.13 ± 0.07	5.71 ± 0.80
43	47.41	2705	Squalene	4.44 ± 0.88	7.32 ± 0.76
47	52.61	2923	Tocopheryl	4.81 ± 0.67	7.00 ± 0.68

## Data Availability

Not applicable.

## Sample Availability

Samples of the compounds are available from the authors.

## References

[1] Tang W.F., Tsai H.P., Chang Y.H., Chang T.Y., Hsieh C.F., Lin C.Y., Lin G.H., Chen Y.L., Jheng J.R., Liu P.C. (2021). Perilla (*Perilla frutescens*) leaf extract inhibits SARS-CoV-2 via direct virus inactivation. Biomed. J..

[2] Zhang Y., Shen Q., Leng L., Zhang D., Chen S., Shi Y., Ning Z., Chen S. (2021). Incipient diploidization of the medicinal plant Perilla within 10,000 years. Nat. Commun..

[3] Ma S.J., Sa K.J., Hong T.K., Lee J.K. (2017). Genetic diversity and population structure analysis in *Perilla frutescens* from Northern areas of China based on simple sequence repeats. Genet. Mol. Res. GMR.

[4] Igarashi M., Miyazaki Y. (2013). A review on bioactivities of perilla: Progress in research on the functions of perilla as medicine and food. Evidence-based complementary and alternative medicine. eCAM.

[5] Hou T., Netala V.R., Zhang H., Xing Y., Li H., Zhang Z. (2022). *Perilla frutescens*: A Rich Source of Pharmacological Active Compounds. Molecules.

[6] Lee J.K., Ohnishi O. (2003). Genetic relationships among cultivated types of *perilla frutescens* and their weedy types in East Asia revealed by AFLP markers. Genet. Resour. Crop Evol..

[7] Ahmed H.M. (2018). Ethnomedicinal, Phytochemical and pharmacological investigations of *Perilla frutescens* (L.) Britt. Molecules.

[8] Makino T., Furuta Y., Wakushima H., Fujii H., Saito K., Kano Y. (2003). Anti-allergic effect of *Perilla frutescens* and its active constituents. Phytother. Res. PTR.

[9] Yu H., Qiu J.F., Ma L.J., Hu Y.J., Li P., Wan J.B. (2017). Phytochemical and phytopharmacological review of *Perilla frutescens* L. (Labiatae), A traditional edible-medicinal herb in China. Food Chem. Toxicol..

[10] Ghimire B.K., Yoo J.H., Yu C.Y., Chung I.M. (2017). GC-MS analysis of volatile compounds of *Perilla frutescens* Britton var. Japonica accessions: Morphological and seasonal variability. Asian Pac. J. Trop. Med..

[11] Seo W.H., Baek H.H. (2009). Characteristic aroma-active compounds of Korean perilla (*Perilla frutescens* Britton) leaf. J. Agric. Food Chem..

[12] Ahmed H.M., Tavaszi-Sarosi S. (2019). Identification and quantification of essential oil content and composition, total polyphenols and antioxidant capacity of *Perilla frutescens* (L.) Britt. Food Chem..

[13] Lee Y.H., Kim B., Kim S., Kim M.S., Kim H., Hwang S.R., Kim K., Lee J.H. (2017). Characterization of metabolite profiles from the leaves of green perilla (*Perilla frutescens*) by ultra high performance liquid chromatography coupled with electrospray ionization quadrupole time-of-flight mass spectrometry and screening for their antioxidant properties. J. Food Drug Anal..

[14] Nakajima A., Yamamoto Y., Yoshinaka N., Namba M., Matsuo H., Okuyama T., Yoshigai E., Okumura T., Nishizawa M., Ikeya Y. (2015). A new flavanone and other flavonoids from green perilla leaf extract inhibit nitric oxide production in interleukin 1β-treated hepatocytes. Biosci. Biotechnol. Biochem..

[15] Fujiwara Y., Kono M., Ito A., Ito M. (2018). Anthocyanins in perilla plants and dried leaves. Phytochemistry.

[16] Kim J.K., Park S.Y., Na J.K., Seong E.S., Yu C.Y. (2012). Metabolite profiling based on lipophilic compounds for quality assessment of perilla (*Perilla frutescens*) cultivars. J. Agric. Food Chem..

[17] Asif M. (2011). Health effects of omega-3,6,9 fatty acids: *Perilla frutescens* is a good example of plant oils. Orient. Pharm. Exp. Med..

[18] Peng Y., Ye J., Kong J. (2005). Determination of phenolic compounds in *Perilla frutescens* L. by capillary electrophoresis with electrochemical detection. J. Agric. Food Chem..

[19] Assefa A.D., Jeong Y.J., Kim D.J., Jeon Y.A., Ok H.C., Baek H.J., Sung J.S. (2018). Characterization, identification, and quantification of phenolic compounds using UPLC-Q-TOF-MS and evaluation of antioxidant activity of 73 *Perilla frutescens* accessions. Food Res. Int. Ott. Ont..

[20] Wang Z., Tu Z., Xie X., Cui H., Kong K.W., Zhang L. (2021). *Perilla frutescens* Leaf Extract and Fractions: Polyphenol Composition, Antioxidant, Enzymes (α-Glucosidase, Acetylcholinesterase, and Tyrosinase) Inhibitory, Anticancer, and Antidiabetic Activities. Foods.

[21] Shin T.Y., Kim S.H., Kim S.H., Kim Y.K., Park H.J., Chae B.S., Jung H.J., Kim H.M. (2000). Inhibitory effect of mast cell-mediated immediate-type allergic reactions in rats by *Perilla frutescens*. Immunopharmacol. Immunotoxicol..

[22] Li Y., Yang X., Chen S., Wu L., Zhou J., Jia K., Ju W. (2021). Integrated Network Pharmacology and GC-MS-Based Metabolomics to Investigate the Effect of Xiang-Su Volatile Oil Against Menopausal Depression. Front. Pharmacol..

[23] Yang J.H., Yoo J.M., Lee E., Lee B., Cho W.K., Park K.I., Yeul Ma J. (2018). Anti-inflammatory effects of Perillae Herba ethanolic extract against TNF-α/IFN-γ-stimulated human keratinocyte HaCaT cells. J. Ethnopharmacol..

[24] Kagawa N., Iguchi H., Henzan M., Hanaoka M. (2019). Drying the leaves of *Perilla frutescens* increases their content of anticancer nutraceuticals. Food Sci. Nutr..

[25] Huang B., Lei Y., Tang Y., Zhang J., Qin L., Liu J. (2011). Comparison of HS-SPME with hydrodistillation and SFE for the analysis of the volatile compounds of zisu and baisu, two varietal species of *Perilla frutescens* of Chinese origin. Food Chem..

[26] Tabanca N., Demirci B., Ali A., Ali Z., Khan I.A. (2015). Essential oils of green and red *perilla frutescens* as potential sources of compounds for mosquito management. Ind. Crops Prod..

[27] Fan Y., Cao X., Zhang M., Wei S., Zhu Y., Ouyang H., He J. (2022). Quantitative Comparison and Chemical Profile Analysis of Different Medicinal Parts of *Perilla frutescens* (L.) Britt. from Different Varieties and Harvest Periods. J. Agric. Food Chem..

[28] Deguchi Y., Ito M. (2020). Rosmarinic acid in *Perilla frutescens* and perilla herb analyzed by HPLC. J. Nat. Med..

[29] Zheng Y.F., Li D.Y., Sun J., Cheng J.M., Chai C., Zhang L., Peng G.P. (2020). Comprehensive Comparison of Two Color Varieties of Perillae Folium Using Rapid Resolution Liquid Chromatography Coupled with Quadruple-Time-of-Flight Mass Spectrometry (RRLC-Q/TOF-MS)-Based Metabolic Profile and *in Vivo/in Vitro* Anti-Oxidative Activity. J. Agric. Food Chem..

[30] Nitta M., Kobayashi H., Ohnishi-Kameyama M., Nagamine T., Yoshida M. (2006). Essential oil variation of cultivated and wild perilla analyzed by GC/MS. Biochem. Syst. Ecol..

[31] Xu Z., Wu W., Zheng Y., Chen L., Cai Q. (2009). Essential oil variations in different Perilla L. accessions: Chemotaxonomic implications. Plant Syst. Evol..

[32] Vogt T. (2010). Phenylpropanoid biosynthesis. Mol. Plant.

[33] Yuba A., Yazaki K., Tabata M., Honda G., Croteau R. (1996). cDNA cloning, characterization, and functional expression of 4S-(-)-limonene synthase from *Perilla frutescens*. Arch. Biochem. Biophys..

[34] Guo L., Zhang D., Wang L., Xue Z., Guo M., Duan L., Zheng Y. (2019). Comparison and Discrimination of *Artemisia argyi* and *Artemisia lavandulifolia* by Gas Chromatography-Mass Spectrometry-Based Metabolomic Approach. J. AOAC Int..

[35] Cebi N., Arici M., Sagdic O. (2021). The famous Turkish rose essential oil: Characterization and authenticity monitoring by FTIR, Raman and GC-MS techniques combined with chemometrics. Food Chem..

[36] Avci A.B., Korkmaz M., Özçelik H. (2014). Essential oil composition of *Cymbocarpum erythraeum* (DC.) Boiss. from Turkey. Nat. Prod. Res..

